# Nutrient provision capacity of alternative livestock farming systems per area of arable farmland required

**DOI:** 10.1038/s41598-021-93782-9

**Published:** 2021-07-22

**Authors:** M. R. F. Lee, J. P. Domingues, G. A. McAuliffe, M. Tichit, F. Accatino, T. Takahashi

**Affiliations:** 1 Harper Adams University, Newport, Shropshire, TF10 8NB UK; 2 INRAE/AgroParisTech, Université Paris-Saclay, 75005 Paris, France; 3 Rothamsted Research, North Wyke, Okehampton, Devon, EX20 2SB UK; 4 University of Bristol, Langford, Somerset, BS40 5DU UK

**Keywords:** Environmental impact, Climate-change mitigation, Nutrition

## Abstract

Although climate impacts of ruminant agriculture are a major concern worldwide, using policy instruments to force grazing farms out of the livestock industry may diminish opportunities to produce nutritious food without exacerbating the food-feed competition for fertile and accessible land resources. Here, we present a new set of quantitative evidence to demonstrate that, per unit of overall nutrient value supplied by a given commodity, the demand for land suitable for human-edible crop production is considerably smaller under ruminant systems than monogastric systems, and consistently so at both farm and regional scales. We also demonstrate that imposition of a naïvely designed “red meat tax” has the potential to invite socioeconomic losses far greater than its environmental benefits, due largely to the induced misallocation of resources at the national scale. Our results reiterate the risk inherent in an excessively climate-focused debate on the role of livestock in human society and call for more multidimensional approaches of sustainability assessment to draw better-balanced policy packages.

## Introduction

Climate impacts arising from ruminant agriculture are a serious concern for humanity^1^. Whilst there are circumstances under which grasslands with grazing livestock can act as a carbon sink^2–4^, such phenomena are generally localised^5^ as well as temporary^6^, and therefore unlikely to have a significant effect at the global scale. The best available information in today’s scientific literature suggests that, when evaluated in carbon dioxide equivalent emitted per unit of food produced, ruminant systems generally emit higher levels of greenhouse gases (GHG) than monogastric livestock systems as well as systems that produce plant-based protein^7–9^.

Nonetheless, to use this evidence to advocate a global dietary shift away from ruminant products^10–12^ creates a curious paradox in light of the ever-growing human population and thus the need to produce more food with less resources. Meeting this demand requires optimal utilisation of farmlands, both cultivated *and* grazed, as it is unlikely that the former can feed future generations on its own^13^. The removal of ruminants does not conform to this principle because, unlike monogastric livestock whose feed is primarily produced on farmlands that are shared with cultivation of human-edible crops, ruminants are able to inhabit grasslands and rangelands^14–16^. Although some of these lands have a capability to produce non-forage crops at a lower yield, a combination of marginal soils, topographies, meteorological conditions and accessibility means that, oftentimes, using ruminants to convert human-indigestible fibre into food that is nutritionally dense and bioavailable is a more sensible option^17–19^. As a recent review on the subject succinctly concluded, “the production of food of animal origin is a very complex process”, of which nuance cannot be understood solely from the GHG perspective^20^.

Here, we present new evidence to demonstrate that per unit of nutrient density scores (NDS)^21,22^, a measure of the overall nutrient value supplied by a food product, the demand for land suitable for human-edible crop production (arable land use: ALU) is considerably smaller under ruminant systems than monogastric systems. This result is robust across multiple datasets encompassing both farm and regional scales. To complement this finding, we also demonstrate that carbon taxation against ruminant production systems has the potential to induce an extremely inefficient resource allocation at the national scale, resulting in socioeconomic losses far greater than its environmental benefits.

## Results

### Farm-scale case study (UK)

The first analysis was conducted using farm-scale data from the UK. Six meat production systems commonly observed in the country were included in the analysis: intensive beef (cereal based), extensive beef (forage based), lowland lamb (grazed on medium-quality soils), upland lamb (grazed on low-quality soils), chicken (indoor) and pork (indoor).

**Figure 1:**
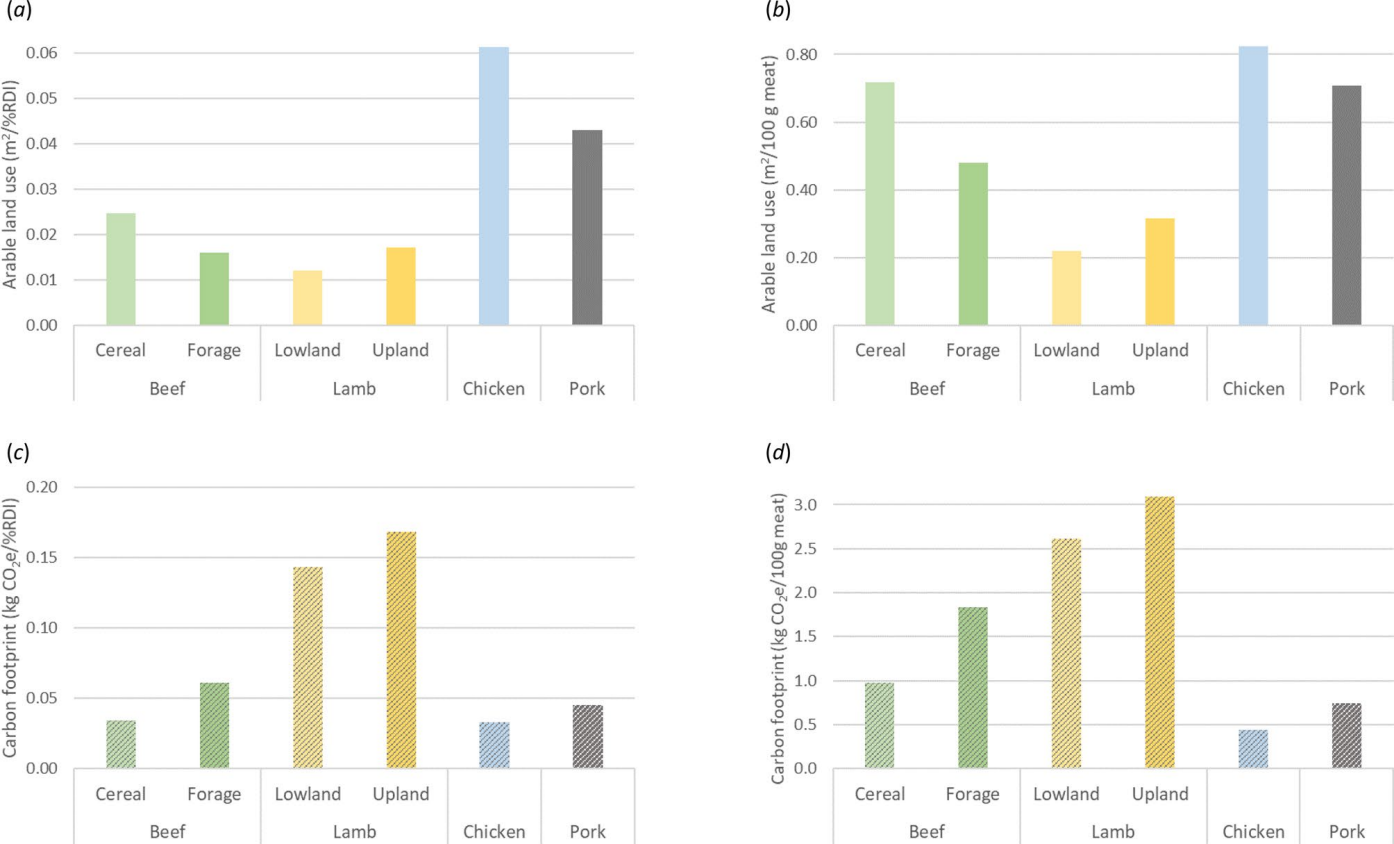
Arable land use per nutrient density score (**a**) and per mass of product (**b**), and carbon footprint per nutrient density score (**c**) and per mass of product (**d**) for six meat production systems commonly observed in the UK. The striking contrast in relative inter-system relationship between the figures demonstrates the challenge facing the sustainability debate surrounding livestock farming. Panels (**c**) and (**d**) were produced from data reported in an earlier study^25^ under the Creative Commons licence CC BY 4.0.

Across the six systems, ALU per NDS—the area (m^2^) required to synthesise 1% recommended daily intake (RDI) for 10 essential nutrients—ranged between 0.012 and 0.061. The smallest area of arable land was required to provide a unit of composite nutrient under lowland lamb, closely followed by forage beef, upland lamb and cereal beef (Fig. 1a). Upland lamb, which carries a better NDS than lowland lamb due to greater contents of nutritionally beneficial long-chain omega-3 polyunsaturated fatty acids (Supplementary Table S1), did not perform as favourably as the lowland system because of the greater need for supplementation with “human-edible feed” per kg liveweight gain to compensate for the lower quality of forages. Nevertheless, the positive overall results for sheep systems are noteworthy in light of the heavy environmental burdens generally associated with the species^23,24^. Pork and chicken systems were shown to occupy up to 3.6 and 5.1 times more arable land than ruminant systems, respectively. The weak performance by monogastric systems here poses a striking contrast to an earlier study^25^, where the same dataset was used to compute the more commonly used metric of carbon footprint per mass of the final product (Fig. 1b–d).

As part of the computational process to derive the above results, the ratio between ALU and NDS was also calculated individually for each component nutrient (Supplementary Table S2). An examination of these values revealed that, with the exception of selenium, of which content is generally lower in ruminant meat than monogastric meat in the UK due to the former’s reliance on pasture grown on low selenium soils^26^, the relative rankings amongst the six farming systems were largely consistent. This finding was further mirrored by the output from the sensitivity analysis using a common alternative NDS formulation with seven essential nutrients (Supplementary Figure S1a), indicating that the results are robust to the choice of nutrients to be included.

### Regional-scale case study (France)

The above case study was conducted with representative farm data, and as such variability in farm size, farming system and productivity within each enterprise (species) is not accounted for. As a means to partially overcome this limitation and evaluate the generality of the farm-scale findings, a second analysis was conducted using regional-scale data from France. The unit of the study was set to be agricultural subregions (*petites régions agricoles*; *n* = 571), with total NDS and total ALU for each subregion estimated across the entire livestock sector. For calculation of ALU, feed production occurring outside the subregion’s geographical boundary (import) was also included to avoid underestimation arising from displacement of local production.

**Figure 2:**
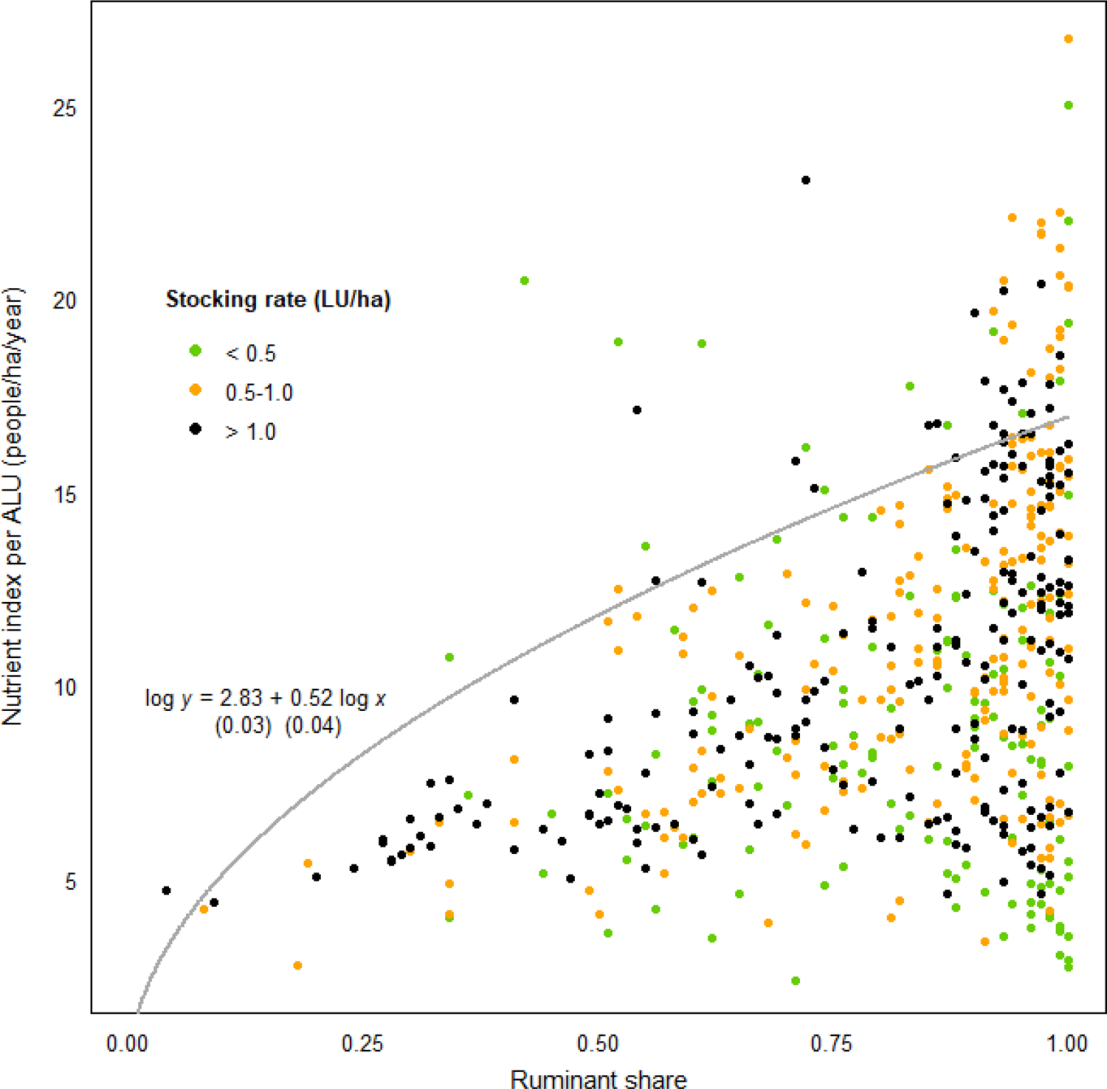
Relationship between the share of ruminants in a subregion’s livestock population and nutrient provision capacity per arable land use (ALU) in France. Each datapoint represents a single agricultural subregion (*petites régions agricoles*), colour-coded by stocking rate. The positive slope of the production frontier function suggests that the greater the ruminant share, the higher the subregion’s potential to provide essential nutrients from a given area of arable land.

The results supported the upward scalability of the findings from the farm-scale case study. A positive association was observed between a subregion’s reliance on ruminants vis-à-vis monogastric livestock, as quantified by the former’s share (0–1) in the total subregional holding of European livestock units^27^, and the subregion’s capability to provide essential nutrients from a given area of arable land (*r* = 0.39, *p* < 0.001) (Fig. 2). This tendency was universally shared across all subregions regardless of their stocking rates but especially strong for those with intensive production systems. A regression analysis found that an increase in the ruminant share (*p* < 0.001) and the interaction term between the ruminant share and the stocking rate (*p* < 0.001) both have positive effects on NDS per ALU.

The relatively weak correlation between the two variables was attributable to the fact that, amongst subregions with a similar level of ruminant share (i.e. amongst datapoints with similar x-values), there is a substantial variability in nutrient production efficiency (i.e. discrepancy in y-values). For example, ~ 15% of subregions with a > 80% ruminant share (at the bottom right cluster in Fig. 2) were found to supply less nutrients per ALU than an average monogastric-focused subregion with a < 50% ruminant share, failing to reduce the food-feed competition. This cluster included subregions under both intensive (high stocking rate) as well as extensive (low stocking rate) farming systems, likely reflecting a high degree of variability in the use of human-edible feed within ruminant livestock systems^28^. However, once this variability was removed using a stochastic frontier analysis^29^, a clearer relationship was observed between the ruminant share and the expected value of NDS per ALU attainable in the absence of inefficient resource utilisation (the concave curve in Fig. 2), with the arable land-saving *potential* of a subregion found to be an increasing function of the ruminant share (*p* < 0.001). Again, these findings were robust to changes in NDS formula (Supplementary Figure S1b).

### National-scale case study (UK and France)

The third analysis was conducted using national scale data from both the UK and France. A computable general equilibrium modelling framework^30^ was developed to estimate the economy-wide impacts of imposing a purchase tax against beef and dairy commodities. The tax rates were set at 18.6% (UK)/19.8% (France) for beef and 11.3% (UK)/12.0% (France) for dairy, drawn from an earlier study that proposed this policy as a means to curb climate change impacts of agri-food systems^31^.

Within the ruminant sector, our results were largely in agreement with the existing literature, most of which are based on partial equilibrium analysis^31–34^. Domestic meat and milk production decreased as expected, with substantial GHG savings recorded both directly on farms and indirectly at connected industries such as manufacturing of agrochemical products (Table 1). Combined together, the proposed tax was forecasted to reduce annual national GHG emissions by 2.5 Mt CO2e (UK)/1.1 Mt CO2e (France).

**Table 1:** Estimated macroeconomic impacts of taxation against the ruminant sector (per year).

Variable	UK	France
Tax rate (meat)	18.6%	19.8%
Tax rate (dairy)	11.3%	12.0%
Domestic production (meat)	− 5.9%	− 1.8%
Domestic production (dairy)	− 0.2%	− 3.5%
GHG savings (on-farm)	1.4 Mt CO_2_e	1.0 Mt CO_2_e
GHG savings (economy-wide)^a^	2.5 Mt CO_2_e	1.1 Mt CO_2_e
Monetised value of economy-wide GHG savings^b^	US$ 129 M	US$ 58 M
Economic welfare losses (equivalent valuation)	US$ 310 M	US$ 232 M
Cost–benefit ratio	2.4	4.0

^a^Includes indirect effects from interconnected industries (e.g. reduced fertiliser production).

^b^Evaluated at US$52/t CO2_e_, the carbon price used to derive the proposed tax rates^31^.

Macroeconomically, however, the countries were predicted to suffer from large welfare losses, primarily caused by forced reallocation of resources such as transfer of land and labour forces from livestock farms to arable farms and non-agricultural industries (Table 1). When converted to the equivalent valuation, a common measure of change in economic welfare solely attributable to policy interventions, the annual losses were predicted to be US$ 310 M (UK)/US$ 232 M (France). Strikingly, these values considerably outweighed the policy benefits of the aforementioned climate change mitigation (US$ 129 M/US$ 58 M), evaluated at the carbon price used to derive the “optimal” tax rates (US$52/t CO2e)^31^. This finding indicates that the current evidence to support carbon taxation may be underestimating the socioeconomic value of ruminants as the best available utiliser of marginal grasslands and rangelands.

## Discussion

The results from the above three case studies collectively elucidate the positive roles ruminant agriculture could play in a wider context of food security. It goes without saying that the current level of red meat consumption in the developed world is unwarranted and detrimental to both environment and human health^35,36^. As such, our finding that ruminant agriculture supplies more essential nutrients per area of arable farmland than monogastric agriculture does not necessarily mean that the sector should be expanded beyond today’s scale. On the other hand, forcing agricultural producers operating on marginal lands to shift away from ruminant production will likely result in forgone opportunities to supply essential nutrients without occupying fertile soils, leading to suboptimal use of global land resources endowed upon us. This may not be a prudent strategy at a time when the demand for livestock products is forecast to increase, and particularly so if we are to address malnutrition and undernourishment at the global scale^37,38^. A “happier medium” must be pursued to balance human nutrition, rural economy and climate change mitigation.

The potential value of non-arable lands in global food production was also demonstrated by a recent study of the US beef sector^39^, which estimated that as much as 43% of the current national supply could be provided from one-half of today’s grassland and human-inedible by-products from arable production systems alone. Thus, under reduced consumption of animal source foods that is widely recommended by medical experts^40^, a combination of ALU saving and by-product utilising ruminant systems could form an integral part of the solution package to meet the demand for nutritionally dense food, especially in light of the strong consumer preference for on-farm practices to reduce environmental footprints^41^. Needless to say, the benefit of this approach must be carefully weighed against climate impacts of maintaining a certain number of ruminants on the planet. Then again, a recent consumer study reported a surprising result that the choice of diet may have little effect on carbon footprint once the issue of overconsumption is accounted for^42^.

The current debate surrounding the role of livestock in human society is primarily focused on GHG emissions, and such prioritisation may well be justifiable given the urgency to tackle climate change. Nonetheless, humanity faces a wide range of environmental, ecological and socioeconomic goals to meet at the same time, both short-term and long-term. Using arable land use and the resultant nutrient provision as a case exemplar, the evidence provided here has reiterated the fact that, at times, different metrics of “sustainability” can result in mutually irreconcilable policy implications that can only be resolved through a comprehensive multidimensional analysis^43^. We contend, therefore, that excessively climate-focused discussions contain a risk of unknowingly creating a suboptimal economy and society, and instead call for more multidimensional approaches of sustainability assessment to draw better-balanced policy packages.

## Methods

### Farm-scale case study (UK)

Under a representative farm approach^25^, the following six meat production systems commonly observed in the UK were considered for this analysis: intensive beef (cereal based), extensive beef (forage based), lowland lamb (grazed on medium-quality soils), upland lamb (grazed on low-quality soils), chicken (indoor) and pork (indoor). For each production system, ALU estimated by an earlier study^28^, originally expressed in the unit of ha/t protein, was converted to m^2^/100 g meat by applying the average protein content reported in the UK food composition table^44^. This value was then divided by NDS, expressed as the percentage of RDI satisfied by 100 g meat, to produce the final metric of ALU per NDS, expressed in the unit of m^2^/%RDI.

The computation of NDS followed the *UK*_*prot*_*10* protocol^25^. Designed specifically for commodities commonly consumed as protein sources, this index gives an equal weight to 10 essential nutrients typically expected from this food group and computes the average percentage of RDI satisfied per unit mass (usually 100 g) of food. The nutrients included in the formula are: protein, monounsaturated fatty acids, long-chain omega-3 polyunsaturated fatty acids, calcium, iron, riboflavin (vitamin B2), folate (B9), cobalamin (B12), selenium and zinc. The adoption of NDS as the study’s functional unit was motivated by the observation that, while ALU has previously been estimated relative to a system’s capability to provide energy and protein^45,46^ as well as a selection of other individual nutrients^47^, such single-nutrient approaches are unable to represent the true value of food as a source of human sustenance^22^ as they fail to internalise nutritional trade-offs between various micronutrients^48^.

The resultant scores represented the average percentage of RDI satisfied across all nutrients by 100 g of an uncooked product. Nutritional compositions of meat produced under the six systems were compiled from frequently cited studies^44,49–52^, whereas RDI values were adopted from UK national recommendations^53^. These data are summarised in Supplementary Table S1. Finally, in order to test the robustness of the relative rankings to the choice of nutrients to be included, the entire process was repeated under the *UK*_*prot*_*7* protocol, an alternative NDS formula that excludes cobalamin, selenium and zinc. Similarly to the *UK*_*prot*_*10* protocol, an equal weight was given to the seven remaining essential nutrients^54^.

### Regional-scale case study (France)

In order to test the upward scalability of findings from the first case study, a second analysis was carried out at a regional scale. Data were collected from 571 agricultural subregions (*petites régions agricoles*) in France that collectively constitute the country’s landmass. Livestock production in each subregion comprised a combination of the following enterprises: intensive beef (cereal based), extensive beef (forage based), dairy cattle, sheep (dual purpose for meat and dairy), goats (dual purpose for meat and dairy), pork, poultry meat (combining chicken, turkey, duck and guineafowl) and laying chickens. Each subregion’s supply of essential nutrients was quantified from subregional output information^27^ and the French national table for nutritional compositions^55^ (Supplementary Table S1). The calculation process accounted for the nutritional value of by-products, for example meat from replaced dairy cows, to consider the full production capacity of farming systems. However, effects of different farming systems on nutritional compositions were not considered based on a recent finding that, relative to inter-commodity differences, these differences are minimal and bear little nutritional implication^56^. The nutrient values were subsequently converted to NDS under the *UK*_*prot*_*10* protocol already used for the farm-scale study but with RDI recommended for the French population^57^ (Supplementary Table S1). The resultant score, initially expressed in the unit of %RDI/day, was subsequently divided by 100 (to convert a percentage to a ratio) and then by 365 (to annualise) to represent the number of people whose demand for essential nutrients can be satisfied for a whole year from the subregion’s annual livestock production.

Each subregion’s ALU across all livestock enterprises was estimated from subregional data on animal population and crop-by-crop acreage under a previously published method^58^. Briefly, the approach first calculates the difference between the local plant resources available as feed and those required by the local population of livestock, and from this value quantifies the area of arable land required outside the subregion’s geographical boundary. As such, the resultant ALU value represents the entire feed produced for the subregion’s livestock, regardless of where it is grown. The present dataset showed that, across 571 subregions, an area equivalent to 76% (6.5 million ha) of the total arable land available in France (8.5 million ha) was used to supply feed for livestock reared in France, through both domestic and overseas production.

The final metric for this analysis, NDS per ALU, was expressed in the unit of people/ha/year and encapsulated the subregion’s nutrient provision capacity per area of arable farmland required. In a manner similar to the farm-scale case study, the entire process was repeated under the *UK*_*prot*_*7* protocol for sensitivity analysis.

### National-scale case study (UK and France)

The third analysis aimed to evaluate national-scale (macroeconomic) consequences of carbon taxation against beef and dairy products, a policy measure suggested by a number of recent studies to reduce GHG emissions associated with ruminant agriculture^31–34^. The idea is based on the results of modelling studies, which predict a weaker consumer demand for taxed products post-intervention. These forecasts, however, were derived under the partial equilibrium framework, a class of economic models exclusively focusing on a single market within an economy—the agri-food market in the present case—under the “separability” assumption that interactions between the studied sector and the rest of the economy are negligible. While this condition is largely satisfied amongst consumers who often set aside a fixed proportion of income for food expenditures^34^, its validity on the production side is less clear. For example, a shrinkage of the ruminant industry could invite knock-on effects on land use and employment structures beyond agriculture, including rural communities that support and depend on business with farmers.

To investigate the extent to which such adverse effects to the macroeconomy may offset the environmental benefit of reduced GHG emissions, a general equilibrium modelling framework was employed. This approach treats all markets in all countries as internal components of the simulation, and therefore does not require the separability assumption described above^59^. More specifically, an uncondensed version of the Global Trade Analysis Project (GTAP) computable general equilibrium model (version 7)^30^ was applied to the GTAP global economic database (version 9a)^60^. The latter’s baseline year was set to 2011. The database, which already incorporates carbon dioxide (CO2) emissions associated with production of commodities and services, was aggregated into 15 sectors and 12 regions to cover the entire global economy (Supplementary Table S3), allowing to internalise the consumer preference between commodities and between origins conditional on exogenously given relative prices. Separately, a global database of non-CO2 GHG emissions^61^ was manually collated into the identical aggregation structure and linked to the main database, so that sector-specific emission factors encompassing all GHGs could be expressed in the unit of t CO2e per quantity of production. The aggregation was conducted in a fashion that would enable disproportionally detailed analysis of agricultural sectors in the UK and France to suit the study’s aim.

Following this process, an *ad valorem* purchase tax against the ruminant sector was separately imposed in each country across independent model runs. The tax rates, outlined in the Results section and also summarised in Table 1, were adopted from a previous study^31^ that used an international carbon price of US$52/t CO2e as a basis to derive these values. In order to avoid double taxation and tax avoidance at the same time, the purchase tax was universally charged against transactions of domestically produced raw materials (cattle carcasses and raw milk) as well as importation of internationally produced raw materials and final products (meat and dairy products). Post-intervention, the modified production level observed in each sector was multiplied by the sector’s emission factor, derived above, to estimate the economy-wide GHG reduction attributable to taxation. Separately, the equivalent valuation for each region was obtained using an existing algorithm^30^ to quantify the welfare impacts brought about by induced reallocation of production resources. Finally, to make these two values comparable against one another, the achieved GHG reduction was monetised under the same carbon price used to derive the tax rates.

## Supplementary Information

Supplementary Information.
