# Supplementary material for: Nutrient provision capacity of alternative livestock farming systems per area of arable farmland required
Source: Sci Rep. 2021 Jul 22;11:14975. doi: 10.1038/s41598-021-93782-9 (PMC8298395; doi:10.1038/s41598-021-93782-9)
Supplement: Supplementary file 1 — Supplementary Information. [file 41598_2021_93782_MOESM1_ESM.pdf]

Nutrient provision capacity of alternative livestock farming systems per area of arable farmland required

MRF Lee[1], JP Domingues[2], GA McAuliffe[3], M Tichit[2], F Accatino[2] and T Takahashi[3,4]*

[1] Harper Adams University, Newport, Shropshire TF10 8NB, UK

[2] INRAE/AgroParisTech, Université Paris-Saclay, 75005 Paris, France

[3] Rothamsted Research, North Wyke, Okehampton, Devon, EX20 2SB, UK

[4] University of Bristol, Langford, Somerset, BS40 5DU, UK

* Corresponding author: taro.takahashi@rothamsted.ac.uk

Supplementary Material (5 pages)

Table S1 Recommended daily intakes and nutrient compositions assumed in farm-scale and regional-scale case studies

Table S2 Arable land use per content of individual essential nutrients

Table S3 Commodity and regional aggregations adopted for national-scale case study

Figure S1 Results of sensitivity analyses using the UK_{prot7} nutrient density scoring system

Table S1. Recommended daily intakes (g) and nutrient compositions (g/100g product) assumed in farm-scale and regional-scale case studies

UK	RDI	Beef cereal	Beef forage	Lamb lowland	Lamb upland	Chicken	Pork
Protein	50.25	23.50	23.50	20.00	20.00	26.30	18.60
MUFA	37.50	1.13	1.63	1.30	1.07	3.70	0.85
EPA+DHA	0.25	0.00	0.03	0.03	0.03	0.02	0.01
Ca	0.700000	0.005000	0.005000	0.012000	0.012000	0.011000	0.010000
Fe	0.011750	0.001600	0.001600	0.001400	0.001400	0.000700	0.000400
Riboflavin	0.001200	0.000260	0.000260	0.000200	0.000200	0.000150	0.000180
Folate	0.000200	0.000016	0.000016	0.000006	0.000006	0.000009	0.000001
Cobalamin	0.000002	0.000002	0.000002	0.000001	0.000001	0.000000	0.000001
Se	0.000068	0.000008	0.000008	0.000003	0.000003	0.000015	0.000011
Zn	0.008250	0.004000	0.004000	0.002000	0.002000	0.001500	0.001300

France	RDI	Beef cereal	Beef forage	Lamb / goat meat	Poultry meat	Pork	Milk (cattle)	Milk (sheep)	Milk (goats)	Eggs
Protein	52.72	24.00	24.00	20.00	20.20	21.00	3.30	5.68	3.22	12.70
MUFA	38.89	1.00	2.00	1.00	4.52	1.00	0.85	1.60	0.71	3.66
EPA+DHA	0.50	0.00	0.03	0.03	0.00	0.02	0.00	0.00	0.00	0.09
Ca	0.900000	0.005000	0.005000	0.010000	0.009690	0.010000	0.117000	0.199000	0.107000	0.076800
Fe	0.012500	0.002000	0.002000	0.003000	0.001630	0.001000	0.000040	0.000460	0.000170	0.001880
Riboflavin	0.001550	0.000300	0.000300	0.000200	0.000480	0.000200	0.000170	0.000340	0.000110	0.000450
Folate	0.000315	0.000020	0.000020	0.000008	0.000010	0.000001	0.000011	0.000009	0.000007	0.000034
Cobalamin	0.000003	0.000002	0.000002	0.000002	0.000001	0.000001	0.000000	0.000001	0.000000	0.000001
Se	0.000055	0.000010	0.000010	0.000005	0.000012	0.000009	0.000002	0.000003	0.000004	0.000003
Zn	0.011000	0.004000	0.004000	0.003180	0.001300	0.001000	0.000370	0.000540	0.000330	0.001010

RDI: recommended daily intake. MUFA: monounsaturated fatty acids; EPA: eicosapentaenoic acid; DHA: docosahexaenoic acid. EPA and DHA are long-chain omega-3 polyunsaturated fatty acids, which are significantly more biologically active than their shorter-chain counterparts [25].

Table S2. Arable land use per nutrient content (m^2/%RDI) computed separately for 10 individual essential nutrients that comprise UK_{prot10} nutrient density score

	Beef_cereal	Beef_forage	Lamb_lowland	Lamb_upland	Chicken	Pork
Protein	0.015	0.010	0.006	0.008	0.016	0.019
MUFA	0.238	0.110	0.064	0.111	0.083	0.311
EPA+DHA	0.527	0.044	0.021	0.025	0.117	0.119
Ca	1.003	0.671	0.128	0.184	0.524	0.495
Fe	0.053	0.035	0.018	0.027	0.138	0.208
Riboflavin	0.033	0.022	0.013	0.019	0.066	0.047
Folate	0.090	0.060	0.073	0.105	0.183	1.414
Cobalamin	0.005	0.004	0.003	0.005	NA	0.011
Se	0.060	0.040	0.050	0.071	0.037	0.043
Zn	0.015	0.010	0.009	0.013	0.045	0.045

RDI: recommended daily intake. MUFA: monounsaturated fatty acids; EPA: eicosapentaenoic acid; DHA: docosahexaenoic acid. NA: Nutrient not detected. EPA and DHA are long-chain omega-3 polyunsaturated fatty acids, which are significantly more biologically active than their shorter-chain counterparts [25].

Within each nutrient, a farming system with a lighter background colour requires a smaller area of arable land to produce 1% RDI.

Table S3. Commodity and regional aggregations adopted for national-scale case study

Commodity [1] (15)	Region (12)
Cattle and other ruminants (live)	UK
Cattle and other ruminants (meat)	France
Raw milk	Rest of the European Union
Milk and dairy products	Oceania
Monogastric animals (live) and eggs	East Asia
Monogastric animals (meat)	Southeast Asia
Grains and Crops	South Asia
Processed Food	North America
Mining and Extraction [2]	Latin America
Textiles and Clothing [3]	Middle East and North Africa
Light Manufacturing	Sub-Saharan Africa
Heavy Manufacturing	Rest of World
Utilities and Construction	
Transport and Communication	
Other Services	

[1] Only listing major commodities within each category
[2] Includes fisheries
[3] Includes wools and other materials of animal origin

(*a*)

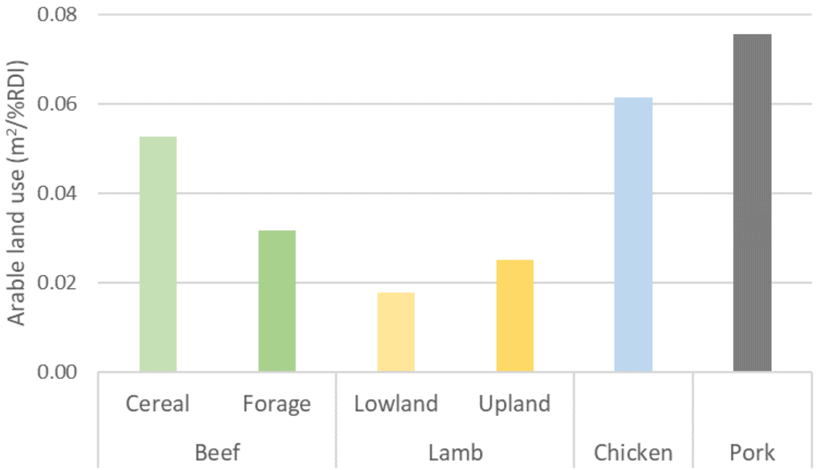

(*b*)

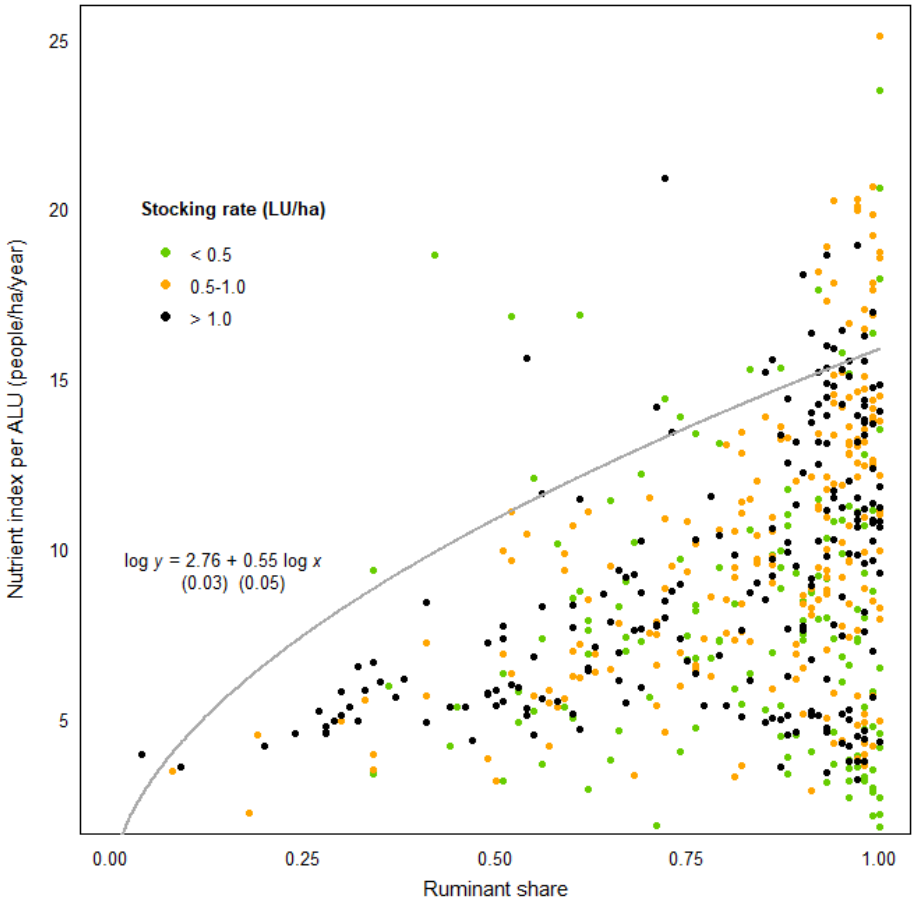

Figure S1. Results of sensitivity analyses using the UK_{prot7} nutrient density scoring system for farm-scale (*a*) and reginal-scale (*b*) case studies. Both panels show similar patterns to the original results derived under the UK_{prot10} system, which are reported as Figure 1a and Figure 2.
